# Challenges in cardiac device innovation: is neuroimaging an appropriate endpoint? Consensus from the 2013 Yale-UCL Cardiac Device Innovation Summit

**DOI:** 10.1186/1741-7015-11-257

**Published:** 2013-12-11

**Authors:** Stephanie M Meller, Andreas Baumbach, Szilard Voros, Michael Mullen, Alexandra J Lansky

**Affiliations:** 1 Yale University School of Medicine and Yale Cardiovascular Research Group, 1 Church Street Suite #330, PO Box 208017, New Haven, CT 06510, USA; 2 The Bristol Heart Institute, University Hospitals Bristol NHS Foundation Trust, Bristol, UK; 3 Stony Brook University Medical Center, Stony Brook, NY, USA; 4 The Heart Hospital, University College London, London, UK

## Abstract

**Background:**

Neurological events associated with transcatheter aortic valve implantation are major contributors to morbidity and mortality. Choosing an appropriate endpoint to determine neuroprotection device efficacy is a key difficulty inhibiting the translation of the innovation from the laboratory to the bedside. Cost and sample size limitations inhibit the feasibility of using the rate of clinical (such as stroke or other cerebral) events as the primary efficacy endpoint. This paper focuses on consensus opinions from the 2013 Yale-University College London (UCL) Device Innovation Summit.

**Discussion:**

Neuroimaging, specifically diffusion-weighted magnetic resonance imaging (DW MRI), may serve as a surrogate endpoint for clinical studies detecting cerebral events in which cost and sample-size limitations prohibit the use of clinical outcomes. A major limitation of using imaging to prove efficacy in cardiac device studies is that no standardized endpoint exists. Ongoing trials investigating cerebral protection devices for transcatheter aortic valve implantation are utilizing and reporting various qualitative and quantitative DW MRI values; however, single lesion volume, number of new lesions, and total lesion volume have been found to be the most reproducible and prognostically important imaging measures.

**Summary:**

DW MRI may be a useful surrogate endpoint for clinical studies detecting cerebral events to determine the device’s success in neurological protection. Consensus from the 2013 Yale-UCL Device Innovation Summit specifically recommends the reporting of mean single lesion volume, number of new lesions, and total volume, and encourages European Union (EU)-US regulatory consensus in the guidance of implementing this endpoint.

## Background

Neurological events contribute to the morbidity and mortality associated with transcatheter aortic valve implantation (TAVI). Neuroimaging, specifically diffusion-weighted magnetic resonance imaging (DW MRI), may be a useful tool for assessing clinical outcomes of both unprotected TAVI as well as in the evaluation of cerebral protection devices.

The current state of neuroprotection for TAVI is of great interest among inventors and clinicians involved in cardiac device development and implementation, and was thus a primary focus of the 2013 Yale-University College London (UCL) Cardiac Device Innovation Summit. This meeting provided a forum for engineers and clinicians to openly discuss the complexities of cardiac embolic protection devices, and address the unmet needs of the regulatory approval process to enhance percutaneous valvular device innovation and clinical implementation.

The 2013 Yale-UCL summit focused on second-generation TAVI, neuroprotection device development and evaluation as adjunct to TAVI, percutaneous mitral valve devices and left ventricular support devices, and novel percutaneous coronary devices including biodegradable stent technologies and targeted biologics. An expert faculty of Europe-based and US-based regulators, industry partners, funders, engineers and clinicians led various discussions throughout the 2-day conference, which was publicized within the Yale and UCL campuses and open to all, with no registration fees. The meeting was sponsored by Yale University and UCL.

Choosing an appropriate endpoint to determine device efficacy is a key difficulty inhibiting the translation of the innovation from the laboratory to the bedside. The Yale-UCL summit developed consensus recommendations regarding the selection of study endpoints, specifically for clinical trials investigating strategies for neuroprotection in TAVI. Below, we will provide a brief discussion of TAVI-related stroke and current strategies for neuroprotection, and provide our conclusions recommending neuroimaging, specifically DW MRI, as a cost-effective and potentially even clinically meaningful endpoint to investigate efficacy of cerebral protection devices for use in cardiac procedures.

### TAVI-related stroke

There are several accepted therapies to treat severe aortic stenosis but each is associated with significant risks. The ‘Placement of AoRTic TraNscathetER’ PARTNER trial and other smaller studies have demonstrated the superiority of TAVI to standard medical therapy for inoperable patients with aortic stenosis and its non-inferiority to surgical valve replacement for high risk patients, with such findings evident up to 2 years post procedure [1,2]. Further implementation of TAVI is limited by the risk of stroke, a devastating contributor to morbidity and mortality in the typically older and relatively frailer patient population undergoing such endovascular procedures.

Indeed, the PARTNER trial demonstrated a two to three times higher risk of stroke with TAVI compared with standard medical therapy or surgery [1], and the rate of TAVI-related stroke is estimated to be between 0% to 11% [3-5], depending on patient and procedural characteristics. The US Food and Drug Administration (FDA) cited the rate of neurological adverse events as a significant concern in approving the Edwards SAPIEN device [6]. Importantly, advances in device technology have led to lower contemporary estimates of periprocedural stroke. In a recent meta-analysis of >10,000 patients, Eggebrecht *et al.* determined the incidence of stroke within the first 24 h of TAVI to be 1.5% ± 1.4%; other studies have found similar results [7,8]. Further, when compared to high-risk surgical cohorts, the rates of complications in TAVI may even be similar to those of surgical valve replacement [9,10]. Improved operator experience and smaller insertion profiles may also decrease the incidence of stroke below that reported in the PARTNER trial [11,12].

The TAVI procedure involves the introduction of bulky devices into atherosclerotic arteries and a calcified aortic valve, and thus lends itself to cerebral embolization of plaque debris. The majority of TAVI-related strokes are in fact periprocedural and >50% occur within the first 24 h of the procedure [1,13]. The cause of periprocedural neurological events during TAVI is probably multifactorial but the pattern of cerebral ischemia following the procedure suggests mechanical embolization of atherosclerotic debris [13,14]. It has been shown that the highest rates of cerebral embolization occur during valve positioning and implantation [15]. Key steps that pose major risk include balloon valvuloplasty, passage of a large-bore catheter, retrograde travel through the aortic arch, and crushing of the native valve leaflets [7]. Hypoperfusion due to rapid ventricular pacing during balloon valvuloplasty or valve implantation is also a possible contributor.

The importance of new lesions found on DW MRI, many of which are clinically silent, remains unclear; however, studies utilizing DW MRI have found new lesions in 58% to 91% of patients undergoing TAVI [15,16]. There is increasing evidence from studies not involving TAVI that the cumulative burden of ischemic brain injury may cause neuropsychological deficits, aggravate vascular dementia, and contribute to cognitive decline [17]. Notably, these studies have shown that the 5-year survival is considerably decreased for patients with vascular dementia compared with age-matched controls (39% versus 75%) [18]. However, though bright lesions on DW MRI are commonly associated with ischemic lesions, they can also be caused by migraines, seizures, or hypoglycemia, and these events may contribute to the positive DW MRI results seen in many patients undergoing TAVI.

The incidence of stroke within 30 days of the TAVI procedure is estimated to be between 1.7% to 6.7%, and there continues to be an increased risk of stroke in the years following the procedure [1,19-21]. Post-procedural neurologic events are likely caused by patient comorbidities such as atrial fibrillation, hypertension and possibly atherosclerotic plaque or thrombus formation at the valve level. Post-procedural DW MRI would have no advantage in detecting or predicting such neurologic events.

Further, Kahlert *et al.* found that 80% of newly detected lesions on DW MRI demonstrated reversal during the 3-month follow-up period; however, apparent lesion reversal does not necessarily mean normalization of brain tissue [15]. In fact, animal studies have shown that even after reversal, neurons exhibit structural damage with histological staining suggesting that other non-neuronal cells may compensate for the alterations in fluid balance [22].

### Adjunctive pharmacology and neuroprotection devices for TAVI

Given the increased risk of stroke associated with TAVI, both adjunctive pharmacotherapy to prevent thrombosis and neuroprotective devices may be indicated. The literature is scarce regarding the appropriate antithrombotic regimen for TAVI, and the few studies that have been conducted have focused both on antiplatelet therapy (including aspirin and clopidogrel) and anticoagulant agents (including bivalirudin and heparin). The only published clinical trial to date randomized 79 patients undergoing TAVI to receive a 300-mg loading dose of clopidogrel on the date of procedure plus post-procedural maintenance therapy consisting of 3 months of 75 mg of clopidogrel daily plus aspirin 100 mg lifetime or aspirin 100 mg alone. The results demonstrated no clinical benefit from the addition of 3 months of clopidogrel maintenance therapy [23]. This finding is important for patients with chronic atrial fibrillation treated with daily warfarin and aspirin, who demonstrate a significantly increased bleeding risk with the addition of clopidogrel for catheterization procedures [24]. Based on the findings of the aforementioned clinical trial, aspirin therapy alone following TAVI is effective and may improve the safety of patients with atrial fibrillation undergoing the procedure. The ‘Effect of BivaliRudin on Aortic Valve Intervention Outcomes’ (BRAVO) 2/3 study will assess the safety and efficacy of using bivalirudin instead of unfractionated heparin in TAVI with the hypothesis that bivalirudin reduces bleeding rates and improves clinical outcomes relative to heparin [25].

In addition to antithrombotic therapy, patients undergoing TAVI may also benefit from the use of cerebral protection devices. The temporal pattern and location of cerebral infarcts and silent ischemic lesions following TAVI indicate periprocedural mechanical embolization as the most likely pathophysiologic mechanism of periprocedural stroke. We thus believe that there is a role for cerebral protection devices in preventing stroke associated with TAVI. The ideal protection device is safe, effective, easy to use, can accommodate various anatomies, demonstrates minimal interference with the TAVI procedure, and importantly, covers all three major cerebral inflow aortic arch vessels. Notably, using protection devices may make the TAVI procedure more cumbersome, complicated, and time consuming, and may thus drive up costs. Results from the ‘Action in Diabetes and Vascular disease: PreterAx and DiamicroN Controlled Evaluation’ (ADVANCE) study link procedural time with the incidence of stroke, suggesting that a fast and simple procedure may be one of the most important factors for stroke prevention (Johan Bosmans, University Hospital of Antwerp, Antwerp, Belgium, personal communication). In addition, lower contemporary stroke rates associated with TAVI raise the question of whether cerebral protection devices and/or adjunctive pharmacotherapy should be recommended for all patients undergoing the procedure. Future randomized controlled trials are needed to determine which patient groups would benefit from these preventative measures. If the increased stroke risk associated with TAVI remains an issue in the future, the implementation of embolic protection devices may be valuable in reducing both clinically evident and occult strokes; however, if further studies suggest that these risks have already been reduced, additional devices may not be warranted.

Current embolic protection devices under clinical investigation include the Edwards Embrella Embolic Deflector (Edwards Lifesciences, Irvine, CA, USA), the Keystone Heart TriGard™ Embolic Deflection Device (Caesarea Business Park, Caesarea, Israel), and the Claret CE Pro™ (Claret Medical, Santa Rosa, CA, USA) (Table 1). While the Embrella and Claret CE Pro are only designed to protect the brachiocephalic and left common carotid arteries, the Keystone Heart device is designed to deflect debris away from all aortic arch cerebral inflow vessels (brachiocephalic, left common carotid, and left subclavian arteries) [26-28].

**Table 1:** Characteristics of current cerebral protection devices for transcatheter aortic valve implantation

**Feature**	**Edwards Embrella Embolic Deflector**[26]	**Keystone Heart TriGard Embolic Deflection Device**[28]	**Claret CE Pro**[27]
Access	Radial	Femoral	Radial
Position	Aorta	Aorta	Brachiocephalic and LCC
Coverage area	Brachiocephalic and LCC	Brachiocephalic, LCC and LSC	Brachiocephalic and LCC
Mechanism	Deflection	Deflection	Capture
Size	6 F	9 F	6 F
Pore Size	100 microns	Approximately 200 microns	140 microns

*LCC* left common carotid artery, *LSC* left subclavian artery.

The only published human study of the Embrella device reports the results of its implantation in three patients undergoing TAVI and one patient undergoing BAV alone. Though no patient developed new neurological symptoms or stroke, a new 5-mm acute cortical infarct was found on predischarge cerebral MRI in the patient who had undergone BAV but remained asymptomatic [26]. Unpublished data of 38 endovascular cases with Embrella implantation from 4 sites in Germany and Canada reported the occurrence of 2 device-related adverse events (1 CVA attributed to malposition of the device, which resolved at discharge; and 1 episode of blurred vision, cause undetermined) and a 2.6% major adverse event rate. Comparison of DW MRI with unprotected historical controls demonstrated similar average numbers of lesions per subject (6.0 versus 4.69 [29] and 3.2 [16]) but a significant reduction in the average volume of lesions in protected subjects versus unprotected historical controls (5.9 cubic centimeters versus 0.394 cubic centimeters [29]) (John G Webb, St. Paul’s Hospital, Vancouver, BC, Canada; personal communication).

Implantation of the Keystone Heart TriGard device in 15 patients resulted in no procedural complications and 1 patient suffering a transient ischemic attack 2 days after the procedure [28]. DW MRI showed 3.2 new cerebral lesions per patient in the study compared with 7.2 lesions per patient in a historical unprotected control group; however, lesion volumes were not reported [28]. In addition, a study involving 40 patients and the Claret CE Pro also revealed no periprocedural incidence of stroke with the device; however, neither DW MRI nor transcranial Doppler were performed. Results from the ongoing DEFLECT I trial will provide DW MRI data in patients undergoing TAVI with the TriGard device in place.

Results of the first in-human studies of neuroprotection devices show promise in reducing the occurrence of neurologic events and thus improving outcomes in TAVI. DW MRI has been used to indicate periprocedural cerebral ischemia after unprotected TAVI in numerous studies [15,29,30]. There are two published studies evaluating the Embrella and TriGard cerebral protection devices, which present the mean or total number of new DW MRI lesions [26,28]; however, ongoing trials are studying mean or total volume of new lesions, as discussed in the following section. The use of various DW MRI endpoints to measure device efficacy implores us to consider if an imaging endpoint is appropriate and if so, then how to define it. The Yale-UCL summit evaluated these important questions and our conclusions are reported below.

## Discussion

### Neuroimaging as an endpoint measure

Neuroimaging modalities, specifically transcranial Doppler ultrasound (TCD) and DW MRI are useful for detecting acute ischemic stroke and have provided additional information about microembolization in TAVI. TCD uses low frequency pulsed sound to allow visualization of the circle of Willis vessels and can identify high-intensity transient signals (HITS) and microembolic signals (MES). Studies have found the detection of HITS in all patients undergoing TAVI and the majority of MES occurring during balloon valvuloplasty and valve delivery [31]. Unfortunately TCD is highly operator dependent and requires considerable skill and experience to attain accurate, reproducible results [32]. DW MRI is used to detect changes in the self-diffusion of water molecules associated with ischemic injury. Given its high sensitivity for detecting brain ischemia and widespread availability, DW MRI is a suitable method for monitoring neurovascular events during interventional procedures [33].

Choosing an appropriate endpoint for a clinical trial can be complex. In fact, between 10% to 15% of medical devices that enter the EU regulatory pathway lack relevant endpoints, which is considered grounds for objection. The penetration rate of devices in general, and in TAVI specifically, is significantly delayed in the US compared to Europe mostly due to FDA requirements for reasonable assurance of safety and effectiveness of a device prior to its approval [28].

For clinical trials investigating neuroprotection devices for use in cardiac procedures, the investigators must prove that the device is able to reduce the occurrence and/or severity of cerebral events. Ideally this would be accomplished by reporting an actual reduction in the rate of stroke, transient ischemic attack, and other neurologic events according to Valve Academic Research Consortium-2 definitions [34]. Because the occurrence of TAVI-related stroke is relatively low (<10%), a large sample size would be needed to detect a difference in clinical event rate with versus without a protection device. In addition to sample size requirements, the rising cost of clinical trials limits the feasibility of using relatively uncommon clinical events as trial efficacy endpoints. Further, silent ischemia accounts for the majority of lesions detected on neuroimaging following TAVI procedures. Using a clinical event endpoint to measure device success would miss the occurrence of these silent lesions, which are associated with cognitive decline and mortality [17,18].

**Figure 1:**
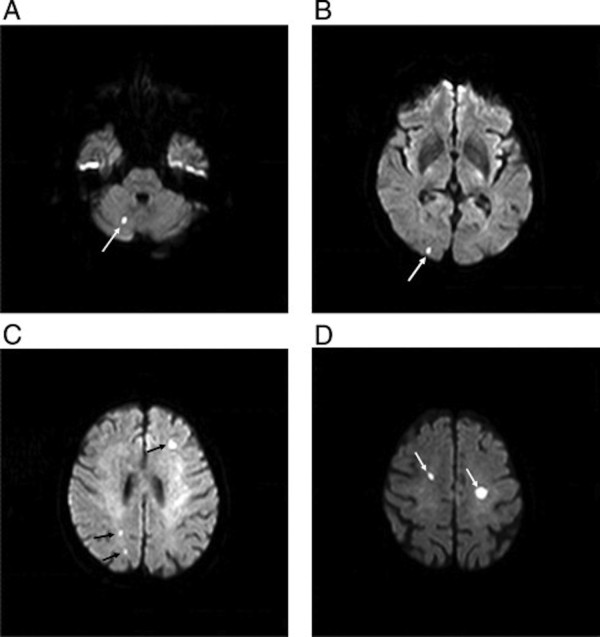
**Diffusion-weighted magnetic resonance imaging (DW MRI) following transfemoral transcatheter aortic valve implantation in an 86-year-old patient.** Multiple acute ischemic lesions in the right cerebellum (**(A)**, white arrow), white occipital territory (**(B)**, white arrow), left frontal and right parietal territories (**(C)**, black arrows), and left and right frontal superior territories (**(D)**, white arrows). Adapted with permission from Rodes-Cabau *et al. J Am Coll Cardiol* 2011, **57:**18–28 [36].

Neuroimaging, specifically DW MRI, may serve as a surrogate endpoint for clinical studies detecting cerebral events in which cost and sample size limitations prohibit the use of clinical outcomes (Table 2). DW MRI, which has sensitivity and specificity up to 92% and 97%, respectively, combines features of conventional spin echo and gradient echo techniques to image the freedom of the diffusion of water molecules to identify restriction in diffusion, suggestive of cerebral ischemia [35]. In cytotoxic edema due to hypoxia, the redistribution of water from the extracellular to the intracellular space is visible within 0 to 5 days of the event (Figure 1). On DW MRI, normal tissue appears gray due to the Brownian motion and diffusion of water molecules, whereas restricted diffusion in the case of ischemia prevents the normal loss of MRI signal and thus appears white. A bright signal on DW MRI and a dark signal on the corresponding apparent diffusion coefficient map is characteristic of acute brain injury within 5 days.

**Table 2:** Clinical trial endpoints that may be used to demonstrate cardiac device efficacy in neuroprotection

**Endpoint measure**	**Advantages**	**Disadvantages**
Incidence of clinical outcomes (such as stroke, transient ischemic attack)	Clear indicator of neurologic events	Low incidence rate demands large sample size to observe effect
	Can be reported in a standardized fashion using the NIH stroke scale and Modified Rankin scale.	Cost limitations may prohibit large sample size
		May miss silent/subtle clinical events
Neuroimaging (such as diffusion-weighted magnetic resonance imaging, transcranial Doppler ultrasound)	Easy and reproducible	No standardized definition of endpoint
	Widely available	Variation in reporting makes cross-study comparisons difficult
		May be contraindicated in some patients (for example, those with pacemakers)
		Radiographic interpretation may be subjective
Biomarkers (such as S100β, apolipoprotein A1, neuron-specific enolase)	Easy	Validity not established
	Reproducible	Normal range for certain patient populations unknown
	Objective	Timing is critical
	Less biased	Expensive
		Subject to laboratory errors

One important issue to consider is that evidence for long-term consequences of lesions detected by DW MRI is lacking. Indeed, recent studies have implied that DW MRI lesions after TAVI are not related to self-sufficiency or mortality 1-year post procedure and that there may even be less cognitive decline post TAVI compared with surgery, despite a higher incidence of embolic lesions [37,38]. These studies are limited by small sample sizes but they suggest that there may limitations in utilizing DW MRI to evaluate TAVI outcomes.

Another major limitation of using DW MRI in clinical trials is that no clear definition of the endpoint exists. Qualitative measurements include lesion number and vascular territory involved and quantitative measurements include total lesion volume, average lesion volume, and maximum lesion volume. All are key neuroimaging endpoint parameters to follow the efficacy of neuroprotection, however, the endpoint must be standardized to allow for cross-study comparison.

Ongoing clinical trials investigating cerebral protection devices for TAVI are utilizing various DW MRI measures to determine device efficacy. The ongoing Prospective Randomized Outcome Study in Patients Undergoing TAVI to Examine Cerebral Ischemia and Bleeding Complications (PROTAVI) trial, which is randomizing patients eligible for TAVI to undergo the procedure with or without the Embrella deflection device, will analyze the rate of new DW MRI brain lesions at 7 days post procedure. Likewise, the DEFLECT I trial is a single arm study enrolling up to 60 patients in the EU, Canada, and Brazil to undergo TAVI with the Keystone Heart TriGard in place using the presence of new DW MRI lesions post procedure compared with a historical control group as a measure of device success.

Although DW MRI lesion presence and rate of occurrence are being used as endpoints, total lesion volume is the most reproducible measurement when performed in an experienced core laboratory, and along with geographic location, provides the best measure of overall burden of ischemic injury, and may therefore be a more appropriate endpoint measure. Though it fails to identify the functional region of the brain involved, studies have identified DW MRI lesion volume as an independent predictor of clinical outcome after acute stroke [39,40]. Specifically, mean lesion volume has been correlated with mental changes and vascular dementia following endovascular procedures [41]. In contrast, the presence and number of DW MRI lesions are only likely to be clinically relevant if the individual lesion is large or in an area of functional significance [42]. Therefore, the Yale-UCL summit concluded that DW MRI lesion volume should be measured by independent core laboratory assessment with validated and reproducible methodology and should be included and reported in all clinical studies using DW MRI to investigate neuroprotection devices for use in TAVI. We recommend that single lesion volume, number of new ischemic lesions, and total lesion volume be measured.

Lastly, in 2011, the FDA issued draft guidance for clinical trial imaging endpoints for studies intending to confirm drug efficacy, recognizing that the use of imaging may assist in the assessment of safety and efficacy as well as patient eligibility. US regulatory requirements have been an impediment to early clinical testing of new devices, which US investigators have mostly outsourced overseas. During the Yale-UCL summit, the FDA expressed its goals to encourage medical device innovation, enhance regulatory science, and facilitate early feasibility clinical studies in the US. Consensus from the Yale-UCL summit called for validation of imaging endpoints in neuroprotection trials involving medical devices and encouraged European regulatory bodies and the FDA to work with the clinical and device industry to support this position.

## Summary

In summary, stroke is a major contributor to morbidity and mortality in TAVI and the development of effective cerebral protection devices may optimize clinical outcomes. Though periprocedural outcomes may already be better than previously thought, it is still necessary to confirm the rates of neurological events in a consistent and reliable manner. Sample size requirements and rising costs of clinical trials are prohibitive to the use of clinical event rates as device efficacy endpoints. The 2013 Yale-UCL Summit developed consensus opinions regarding this topic. DW MRI may be a sensitive and specific surrogate endpoint for clinical studies detecting cerebral events to determine the device’s success in neurological protection; however, further research is needed. Finally, for clinical trial investigators using DW MRI as an endpoint to detect cerebral events, we recommend the reporting of mean single lesion volume, number of new lesions, and total volume, as we have concluded that these values are the most reproducible and potentially even prognostically meaningful DW MRI measures.

## Abbreviations

BAV: Balloon aortic valvuloplasty; CVA: Cerebrovascular accident; DW MRI: Diffusion-weighted magnetic resonance imaging; EU: European Union; FDA: US Food and Drug Administration; TAVI: Transcatheter aortic valve implantation; UCL: University College London.

## Competing interests

The authors are investigators in the ongoing DEFLECT I trial (Keystone Heart, Ltd; Herzliya, Israel).

## Authors’ contributions

All authors have met the full criteria and requirements for authorship. SMM contributed in the conception and design of the manuscript as well as drafting of the manuscript. AB supervised drafting of the background section, ‘Review of adjunctive pharmacology and neuroprotection devices for TAVI’ and SV supervised drafting of the sections involving neuroimaging. AJL and MM directed and led the Yale-UCL Summit, including the sessions discussed in this article. All authors contributed in revising the manuscript critically for intellectual content. All authors have provided final approval of the manuscript submitted.

## Authors’ information

AJL and MM organized and led the 2013 Yale-UCL Cardiac Device Innovation Summit, which the remaining authors participated in. AJL is an associated Professor of Cardiology at Yale University School of Medicine and directs the Yale Cardiovascular Research Group (YCRG) and the Yale Valve Program. MM is a consultant cardiologist at the Heart Hospital, University College London, and leads the Structural Heart Intervention program. SMM is a medical student at Yale University School of Medicine conducting research with AJL at YCRG. AB is a consultant cardiologist at University Hospitals Bristol in the UK and heads clinical research in the Department of Cardiology there. SV is an Associate Professor of Medicine/Cardiology and Radiology, and Director of Advanced Cardiovascular MR and CT Research at the Department of Radiology and Cardiology at Stony Brook University Medical Center.
